# Use of FRET-Sensor ‘Mermaid’ to Detect Subtle Changes in Membrane Potential of Primary Mouse PASMCs

**DOI:** 10.3390/cells13121070

**Published:** 2024-06-20

**Authors:** Ruth C. Dartsch, Simone Kraut, Tim Mayer, Andreas Gabel, Alexander Dietrich, Norbert Weissmann, Beate Fuchs, Fenja Knoepp

**Affiliations:** 1 Cardiopulmonary Institute (CPI), Universities of Giessen and Marburg Lung Center (UGMLC), Member of the German Center for Lung Research (DZL), Justus-Liebig-University, 35392 Giessen, Germany; 2 Walther-Straub-Institute for Pharmacology and Toxicology, Member of the German Center for Lung Research (DZL), Ludwig-Maximilians University, 80539 Munich, Germany; mail@tim-mayer.eu (T.M.);

**Keywords:** FRET sensor, Mermaid, PASMC, membrane potential, pulmonary vasculature

## Abstract

Subtle changes in the membrane potential of pulmonary arterial smooth muscle cells (PASMCs) are pivotal for controlling pulmonary vascular tone, e.g., for initiating Hypoxic Pulmonary Vasoconstriction, a vital mechanism of the pulmonary circulation. In our study, we evaluated the ability of the fluorescence resonance energy transfer (FRET)-based voltage-sensor Mermaid to detect such subtle changes in membrane potential. Mouse PASMCs were isolated and transduced with Mermaid-encoding lentiviral vectors before the acceptor/donor emission ratio was assessed via live cell FRET-imaging. Mermaid’s sensitivity was tested by applying specific potassium chloride (KCl) concentrations. These KCl concentrations were previously validated by patch clamp recordings to induce depolarization with predefined amplitudes that physiologically occur in PASMCs. Mermaid’s emission ratio dose-dependently increased upon depolarization with KCl. However, Mermaid formed unspecific intracellular aggregates, which limited the usefulness of this voltage sensor. When analyzing the membrane rim only to circumvent these unspecific signals, Mermaid was not suitable to resolve subtle changes in the membrane potential of ≤10 mV. In summary, we found Mermaid to be a suitable alternative for reliably detecting qualitative membrane voltage changes of more than 10 mV in primary mouse PASMCs. However, one should be aware of the limitations associated with this voltage sensor.

## 1. Introduction

Hypoxic Pulmonary Vasoconstriction (HPV) is the intrinsic mechanism governing the pulmonary circulation’s response to lowered oxygen (O_2_) concentrations [1,2,3,4,5]. Via the finely tuned vasoconstriction of precapillary arteries in response to localized hypoxia, HPV matches perfusion to ventilation, thereby maintaining efficient pulmonary gas exchange and thus optimal oxygenation of the blood [1,5,6]. A disturbed HPV, as often observed under pathological conditions, such as pneumonia, acute lung injury (ALI), acute respiratory distress syndrome (ARDS) [1,7] or COVID-19 [8,9,10] lead to inadequate oxygenation of the blood, resulting in life-threatening hypoxemia. However, the detailed cellular mechanisms underlying this important physiological mechanism are still incompletely understood [11,12].

It is widely accepted that the sensor and effector cells of HPV are precapillary pulmonary arterial smooth muscle cells (PASMCs) [1]. In particular, the membrane potential of these cell types plays a crucial role in initiating HPV: The membrane potential of PASMCs depolarizes upon alveolar hypoxia [1,3,13,14,15,16,17], leading to an influx of Ca^2+^ ions consecutively inducing smooth muscle cell contraction and thus HPV initiation. In contrast, therapeutic strategies aimed at hyperpolarizing cellular membrane potential, e.g., inhaled nitric oxide (NO), trigger vasodilation of pulmonary arteries [18]. Consequently, understanding the intricate relationship between PASMC membrane potential and cellular contractility is essential in deciphering the mechanisms underlying pulmonary vascular responses.

Since its discovery in the 1980s, the gold standard methodology for elucidating changes in cellular membrane potential is still the patch clamp technique [19,20] that provides superior temporal accuracy and sensitivity [20]. However, despite the indisputable power of patch clamp recordings, this technique comes with some challenges and limitations [20], particularly because of the labor-intensive and time-consuming nature of this highly sophisticated technique, patch clamp is not available in every laboratory.

A promising alternative to depict changes in membrane potential are represented by optical sensors, such as chemical dyes or genetically encoded voltage indicators (GEVIs) [21]. GEVIs are based on an integral membrane protein and provide numerous advantages, including the ability to target the sensor to specific cell types by selecting cell-specific promoters or by enabling multicolor images [22]. They are constructed using either a single fluorophore or a pair of fluorescence resonance energy transfer (FRET) fluorochromes [22,23,24,25]. In such (FRET) sensors, two different-colored fluorochromes, donor and acceptor, are fused in tandem to a voltage-sensitive transmembrane protein, such as the voltage-sensing domain of the *Ciona intestinalis* voltage-sensing phosphatase (Ci-VSP) [26]. Similar to voltage-gated ion channels, the voltage-sensing domain of Ci-VSP is composed of four transmembrane segments, with positively charged amino acids periodically interspersed with hydrophobic residues in the S4 segment [26]. Based on this voltage-sensing domain, in 2008, Tsutsui and co-workers developed a genetically encoded FRET-sensor that was optimized for direct visualization of electrical activities in living cells, which they named Mermaid [24]. In Mermaid, the voltage-sensing domain of Ci-VSP is fused to two coral derived fluorescents proteins in tandem, namely monomeric Umi-Kinoko (mUKG, donor) and an optimized version of monomeric Kusabira orange (mKOκ, acceptor), respectively. Alterations in membrane potential elicit a conformational change in the voltage-sensing domain [26], thus altering the spatial relationship between the two fluorophores, consequently modifying the efficiency of FRET between mUKG and mKOκ. The ratiometric readout of the fluorescence intensities between donor and acceptor then serves as a relative readout for changes in cellular membrane potential [27].

Since its discovery, Mermaid has been evaluated on only a few cell types, mainly neuronal [24,28,29] and cardiac cells [30,31]. As excitable cells, both of these cell types are characterized by strong changes in the membrane potential, i.e., action potentials with an amplitude ranging between 60 and 120 mV [32]. These strong signals could be reliably detected by Mermaid.

In contrast to excitable cells, the physiological changes in membrane potential of PASMCs are far smaller, with an amplitude between 5 and 15 mV, e.g., in response to hypoxia and NO, respectively [2,3,18,33]. In addition, the use of Mermaid in pulmonary cell types, PASMCs in particular, has not yet been reported. Since Mermaid might represent a feasible alternative whenever classical patch clamp approaches are not available, we aimed to test whether this FRET sensor (i) is applicable in primary mouse PASMCs and (ii) allows reliable measurements of subtle membrane voltage changes in these cells.

## 2. Materials and Methods

### 2.1. Primary PASMC Isolation and Cell Culture

Primary mouse precapillary arterial smooth muscle cells were isolated from precapillary pulmonary arterial vessels of C57BL/6J mice according to a previously published protocol [2,34,35]. Cell isolation was approved by the ‘Institutional Animal Investigation Care and Use Committee’ and the appropriate governmental committee (GI20/10 No. A28/2009, No. A34/2011 and G70/2019). PASMCs were isolated from three to four individual mice and cells were cultured at 37 °C, 5.3% CO_2_ for 5–6 days on sterilized glass coverslips (for Mermaid measurements) or 35 mm cell culture dishes (for patch clamp recordings) in Smooth Muscle Cell Growth Medium 2, (PromoCell, Heidelberg, Germany) supplemented with 15% fetal calf serum (FCS) and 1% penicillin/streptomycin. All measurements were performed with unpassaged cells (P0).

Human embryonic kidney cells (HEK 293T cells, DSMZ, ACC 635, Braunschweig, Germany) for lentiviral vector production were maintained in Minimum Essential Medium (Invitrogen, Karlsruhe, Germany) supplemented with 10% FCS and 1% penicillin/streptomycin at 37 °C in a 21% O_2_ and 5.3% CO_2_ humidified atmosphere.

### 2.2. HEK Cell Culture, Lentivirus Production and PASMC Infection

Lentiviruses were produced according to a previously described protocol [36] with slight modifications as follows: the pH of the 2× HBS- and 0.1× TE-buffer were adjusted to 7.05 and 7.6, respectively. Virus production and transduction of primary cells were carried out according to S2 safety regulations and were approved by the local authorities.

The Mermaid-coding transfer plasmid [24] was generously provided by Tsutsui et al. and subcloned in pWPXL (Addgene Plasmid #12257). Therefore, the Mermaid-encoding sequence was excised with BamHI and EcoRI and ligated into pWPXL (instead of GFP) between positions 3499 and 4316, respectively.

Afterwards, a 2nd-generation lentivirus system consisting of the packaging plasmid (psPAX2, Addgene Plasmid #12260), a VSV-G coding envelope plasmid (PMD2.G, Addgene Plasmid #12259) as well as the Mermaid-encoding transfer plasmid were transduced into HEK293T cells by calcium phosphate precipitation. Lentivirus-containing supernatants were harvested every 12 h 2–3 times, pooled and centrifuged at 1500 rpm (centrifuge Rotina 420 R with Rotor 4790-A, Hettich, Tuttlingen, Germany) for 5 min, filtered with 0.22 µm and stored at 4 °C for immediate use or at −80 °C for long-term storage. Unconcentrated supernatants were used for infection. In brief, primary PASMCs were transduced with 4 mL of lentivirus containing a supernatant per well of a six-well plate and supplemented with 8 µg/mL polybrene (Hexadimethrinbromid, Millipore, Schwalbach, Germany). Transduction was stopped after 6 h by media change. FRET imaging experiments were carried out 3 days post transduction.

### 2.3. FRET Imaging

Live cell imaging experiments were carried out with an Olympus IX-71 inverted microscope (Olympus, Hamburg, Germany), equipped with a 150 W xenon lamp (Polychrome 5, TILL Photonics, Gräfelfing, Germany). The FRET sensor was excited at 460 nm with an exposure time of 50 ms throughout all experiments. Images were taken at 0.5 Hz for 10 min. The emitted fluorescence signal was splitted with a beamsplitter at 565 nm (Optical insights Multispec 565, AHF Analysentechnik, Tübingen, Germany). Donor and acceptor emission passed emissions filters (BrightLine HC 520/35 and BrightLine HC 585/40, AHF Analysentechnik, Tübingen, Germany) and was captured on a CCD camera. All experiments were carried out at 33 °C according to [24].

Coverslips were transferred into an open and non-perfused microscope chamber and covered with Hepes Ringer (5.6 mM KCl, 136.4 mM NaCl, 1 mM MgCl_2_, 2.2 mM CaCl_2_, 11 mM Glucose, 10 mM Hepes, pH 7.4 ± 0.01 adjusted with NaOH). KCl was applied by manual pipetting in indicated concentrations 1 min after the start of the experiment.

### 2.4. Direct Immunofluorescence and Confocal Microscopy

For α-smooth muscle actin staining, unpassaged primary PASMCs were grown in an 8-well chamber slide for 6 days and fixed with 1:1 aceton–methanol for 5 min, washed and blocked with 3% bovine serum albumin in Dulbecco’s Phosphate Buffered Saline (DPBS, PAN Biotech, Aidenbach, Germany) for 45 min and incubated with a Cy3™ coupled α-smooth muscle actin antibody (Sigma-Aldrich, München, Germany) in 0.1% BSA in DPBS (1:400, Sigma-Aldrich, Steinheim, Germany) for 60 min at room temperature. DAPI (Sigma-Aldrich, Steinheim, Germany) in a final concentration of 1 µM was used for nuclear counterstaining. Coverslips were mounted with a DAKO fluorescent mounting medium (Dako cytomation, Hamburg, Germany). Pictures were taken with a confocal laser scanning microscope (Leica TCS SP5 X, Leica Microsystems, Wetzlar, Germany). Cy3™ and DAPI were excited with a 546 nm laser and a 405 nm laser, respectively.

### 2.5. Data Analysis of FRET Measurements

FRET measurements were performed using TILLVisION software (Version 4.0, TILLPhotonics GmbH, Grafelfing, Germany), while subsequent analyses were conducted with ImageJ (Version 1.45, National Institutes of Health, Bethesda, MD, USA). Raw images were exported from TILLVisION as *.tiff files and imported into ImageJ as an image sequence before being converted into a 32-bit format and adjusted for brightness and contrast. Splitting of the combined donor/acceptor image was achieved using the freely available plugin ‘OIcut RGBmerge’. The splitted image halves were stored for further analyses as separate files. To correct for a discrete image misalignment of donor and acceptor overlay and to enable final exact ratio calculation, donor image was adjusted via ‘Image > transform > translate’ to an exact overlap with the acceptor image. Translation of donor image was verified via subtraction of acceptor channel minus donor image using the ‘Process > Image calculator’ command.

Subsequently, background correction was performed using the freely available Makro ‘BG Subtraction from ROI’. After defining a background ROI (region of interest), background fluorescence was subtracted from the corresponding image sequence. Correction was performed for acceptor as well as donor image sequences separately. To enable ratio calculation, the thresholds for donor and acceptor sequences were adjusted via ‘Image > Adjust > Threshold’ so that only cells were identified as fluorescent. Background fluorescence was cut via set background pixels to ‘NaN’.

The ratio was then calculated via ‘Process > Image calculator: acceptor divide donor’. The resulting ratio was finally stored as a separate *.tiff file.

For generating membrane-only image sequences, cell shapes of the previously calculated ratio image sequences were eroded by ‘Process > Binary > Erode’. For this, the according ratio image stack was duplicated and termed ‘1’, threshold was again adjusted, and the background was set to black via ‘black background’. The adjusted image was then duplicated again, termed ‘1-1’. The cellular shape in duplicated image ‘1-1’ was then eroded three times by ‘Process > Binary > Erode’. The membrane-only image stack was processed via the subtraction of both duplicate images: ‘Process > image calculator: 1 subtract 1-1’. To finally generate the fluorescence intensity, the image was multiplied with the membrane-only image via ‘Process > Image Calculator > Multiply’.

For data analysis, the fluorescence intensity values were depicted via ‘Stacks > plot z-axis profile’ and copied to MS Excel (Version 15.0, Microsoft Corporation, Redmond, WA, USA).

Each cell’s raw fluorescence data were normalized by setting the first value of each individual cell to 100%. Only cells with a threefold higher fluorescence intensity of the donor compared to the background fluorescence were used for analyses. As commonly observed, a very small number of cells died during the multi-day cultivation procedure or in response to lentiviral transduction. In extremely isolated cases, cell death occurred after expression of Mermaid, leaving a fluorescent but dead cell that was morphologically indistinguishable from living cells. Although fluorescent, these occasionally occurring cells were characterized by the absence of a response to extracellular KCl, which is a common feature of all living cells. To ensure that the results were not compromised by the presence of dead cells, which are sporadically but inevitably present in the culture dish, we implemented a quality control measure by including only those cells in the analysis that were characterized by a simultaneous decrease in donor fluorescence and increase in acceptor fluorescence (for FRET measurements) or a negative membrane potential plus a KCl-induced depolarization (in patch clamp experiments).

For statistical analysis of FRET ratio changes between different potassium chloride concentrations, five consecutive values directly before KCl application (−KCl) were averaged and normalized to the average of five consecutive values taken 2 min after KCl application (+KCl) by subtracting FRET ratio (+KCl) minus FRET ratio (−KCl). Values are shown as ∆ FRET ratio (%) ± SEM.

### 2.6. Cell Harvesting for Patch Clamp Recordings

All media for cell harvesting were prewarmed to 37 °C. On the day of the experiment, cells were washed twice with ROTI^®^Cell PBS/EDTA (Carl Roth, Karlsruhe, Germany). After the removal of PBS/EDTA, Trypsin-replacement enzyme TrypLE^TM^ (ThermoFisher, Darmstadt, Germany) was added, and cells were incubated for 5 min at 37 °C in an incubator (5.3% CO_2_). Afterwards, cells were gently resuspended in a 50:50 mixture consisting of an extracellular solution without Ca^2+^/Mg^2+^ (External [-] Ca^2+^ [-] Mg^2+^, Nanion Technologies, Munich, Germany) and Dulbecco′s Modified Eagle′s Medium/Nutrient Mixture F-12 Ham (Sigma Aldrich, St. Louis, MO, USA) before being transferred to the Patchliner’s cell hotel (prewarmed to 25 °C) where they were periodically resuspended to minimize clustering. After a short waiting period of 5–10 min, cells were used for automated patch clamp recordings.

### 2.7. Solutions

The extracellular solution contained (in mM) 141 NaCl, 5.6 KCl, 2.2 CaCl_2_, 1 MgCl_2_, 11 Glucose and 10 Hepes (pH 7.4), while the intracellular solution (Internal KF110, Nanion Technologies) consisted of (in mM) 110 KF, 10 NaCl, 10 KCl, 10 Hepes and 10 EGTA, supplemented with 5 Mg-ATP (pH 7.2). In accordance with Wilson et al., 15 mM NaCl was added to the intracellular solution in order to prevent unwanted depolarization of membrane potential resulting from the inward current through the seal resistance, thus allowing for accurate measurement of the resting membrane potential [37].

For assessing changes in membrane potential, KCl was added to the extracellular solution to achieve a total concentration of 25 mM, 50 mM and 100 mM KCl.

### 2.8. Assessment of Cellular Membrane Potential via Automated Patch Clamp Recordings

Automated whole-cell patch clamp recordings were conducted in the current clamp configuration (I = 0) using the Patchliner Octo System (Nanion Technologies, Munich, Germany) that was coupled to two QuadroEPC-10 USB amplifiers (HEKA Elektronik, Lambrecht/Pfalz, Germany). Cell catch, seal formation, whole-cell access and conduction of up to eight simultaneous experiments was automatically controlled by the PatchControlHT software (Version 3.01.22, Nanion Technologies, Munich, Germany). Patchmaster software (Version v2x92, HEKA Elektronik, Reutlingen, Germany) was used for data acquisition, while data analysis was performed via Fitmaster (Version v2x92, HEKA Elektronik, Reutlingen, Germany) and GraphPad Prism 9 (GraphPad Software Inc., San Diego, CA, USA). Borosilicate glass NPC-16 chips (high resistance, single-use, disposable; Nanion Technologies) were used for patch clamp recordings.

Cells were transferred from the cell hotel to each chip well and caught by suction that was applied via the internal pressure controller. Once stable whole-cell configuration was reached (seal-resistance ≥ 1 GΩ), the experimental protocol was initiated. Liquid junction potential (11.6 mV) was corrected manually during analysis. Currents were low-pass filtered using the internal Bessel filter of the EPC-10 and sampled at 2.5 kHz.

### 2.9. Statistical Analysis

Statistical analysis was carried out using GraphPad Prism 9 (GraphPad Software Inc., San Diego, CA, USA) by means of one-way ANOVA and uncorrected Fisher’s least significance difference (LSD) test. Data are shown as mean ± SEM, while the exact group size is stated in each figure legend. Results were considered significant with *p* values < 0.05.

## 3. Results

### 3.1. Validation of Mermaid- Expression in Primary PASMCs and Experimental Setup

**Figure 1:**
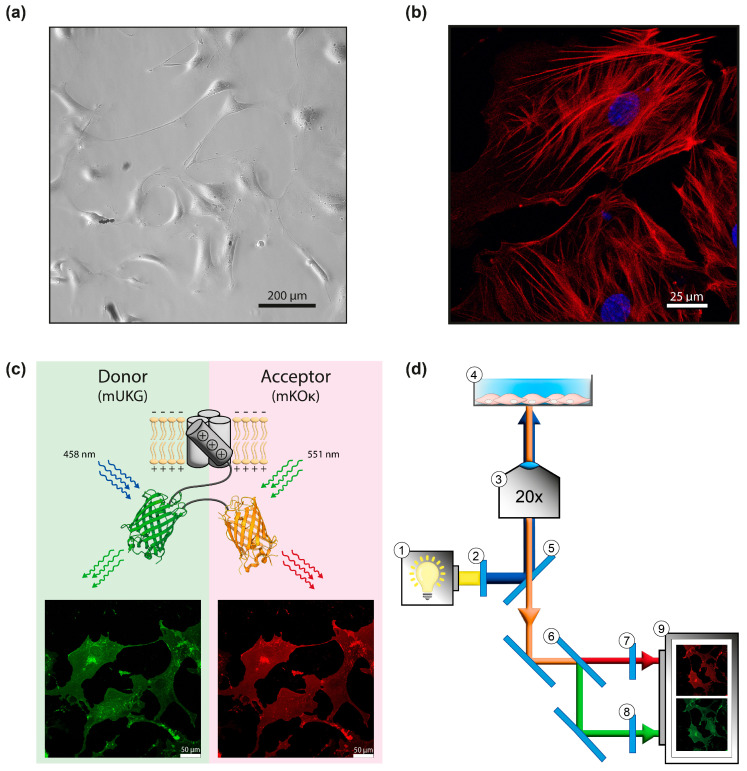
Validation of Mermaid expression in primary mouse PASMCs and experimental setup. (**a**) Phase contrast image of primary mouse PASMCs, scale bar 200 µm. (**b**) Confocal image of mouse PASMCs stained against α-smooth muscle actin (red) and nuclear counterstain (DAPI, blue), scale bar 25 µm. (**c**) Schematic illustration showing Mermaid expression with individually taken confocal images of donor emission (mUKG, green: emission filter 470–540 nm, left hand side) excited at its excitation maximum at 458 nm as well as acceptor emission (mKOκ, red: emission filter 568–587 nm, right hand side) excited at its excitation maximum at 551 nm, scale bar 50 µm. (**d**) Schematic illustration of the optical configuration for simultaneous detection of donor and acceptor fluorescence signals in FRET live cell imaging: ① 150 W Xenon lamp, ② excitation filter (BrightLine HC 472/30), ③ objective (20×, Olympus, UPlanSApo, 20×/0.85 Oil), ④ Mermaid-expressing PASMC covered with Hepes Ringer, ⑤ beamsplitter BS 495, ⑥ beamsplitter Optical insights Multispec 565, ⑦ emission filter (BrightLine HC 585/40), ⑧ emission filter BrightLine HC 520/35 ⑨ CCD camera. The beam path within the setup is represented by arrows.

To check whether Mermaid represents a suitable sensor to observe changes in membrane potential in primary mouse PASMCs, cells were freshly isolated from mouse precapillary pulmonary arteries and cultured for 5–6 days (Figure 1a). After this period, unpassaged (P0) primary mouse PASMCs exhibited a strong cytoplasmatic fluorescence signal when stained against α-smooth muscle actin, a SMC-specific marker (Figure 1b).

PASMCs were transduced at day 5–6 post isolation. Three days later, Mermaid expression and fluorochrome functionality were validated by confocal microscopy. Therefore, donor (mUKG) and acceptor fluorochrome (mKOκ) were excited separately at their excitation maxima of 458 nm and 551 nm, respectively. Emission of each fluorochrome (470–540 nm for donor and 568–587 nm for acceptor, respectively) showed a robust fluorescence signal (Figure 1c and Appendix A, Figure A2) in fixed PASMCs. Having confirmed the expression of Mermaid in primary PASMCs, live cell FRET experiments were conducted on an inverted microscope equipped with a Xenon lamp as shown in Figure 1d. Upon excitation at 460 nm, the emitted photons were split via beamsplitters to simultaneously detect donor and acceptor signals. After passing their respective emission filters, fluorescence signals were finally detected and digitalized by a CCD camera (Figure 1d).

### 3.2. Functional Validation of Mermaid in Primary Mouse PASMCs

**Figure 2:**
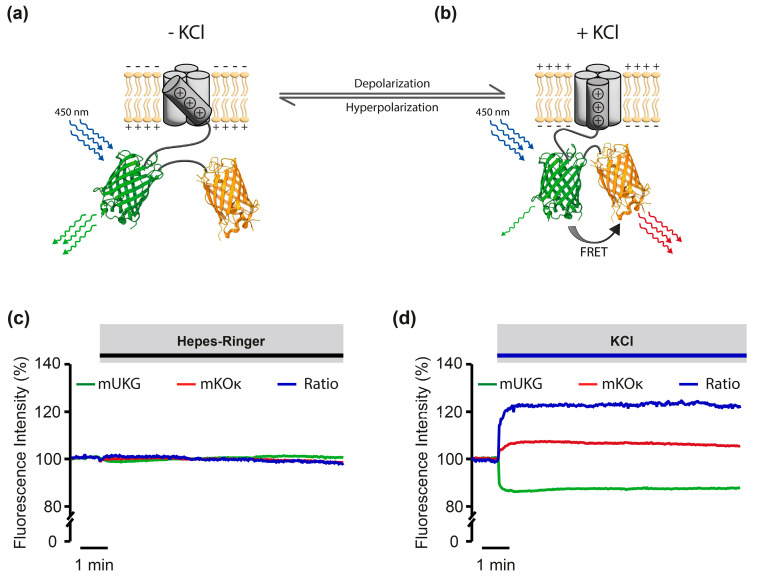
Validation of Mermaid’s functionality in primary mouse PASMCs. (**a**) Schematic showing Mermaid in the absence of potassium chloride (−KCl). The two coral-derived fluorescence proteins mUKG (green, donor) and mKO_K_ (orange, acceptor) are coupled to the transmembrane voltage sensor. Donor emission (green curly arrows) is induced upon excitation at 450 nm, while acceptor emission is absent under these conditions. (**b**) Mermaid after application of KCl (+KCl). The KCl-mediated depolarization induced a conformational change that brings donor and acceptor fluorochromes into proximity. The convergence of the two fluorochromes enables the energy transfer from donor to acceptor (black arrow, FRET), thus resulting in the simultaneous decrease in donor fluorescence emission (single green curly arrow) and enhanced acceptor fluorescence emission (red curly arrows). (**c**) Representative FRET measurement of a single cell in the absence of KCl. Donor fluorescence (mUKG) intensity is shown in green, while the acceptor fluorescence intensity (mKO_K_) is colored in red. The corresponding acceptor/donor ratio is highlighted in blue. As control, the cell was challenged with Hepes Ringer, depicting no changes in fluorescence emission. (**d**) Donor fluorescence intensity (green), acceptor fluorescence intensity (red) as well as acceptor/donor ratio (blue) of a single cell challenged with potassium chloride (KCl), depicting an increase in acceptor fluorescence emission and a decrease in donor fluorescence emission as well as a concomitant increase in FRET ratio.

Since the fluorescence images alone do not prove Mermaid’s localization in the plasma membrane of infected PASMCs beyond doubt, we additionally tested its functionality based on its response to membrane voltage changes. Therefore, Mermaid-expressing cells were exposed to extracellular potassium chloride (KCl), a well-established stimulus to induce cellular depolarization: an elevation in extracellular K^+^-concentration diminishes the magnitude of the K^+^-gradient across the cell membrane, thereby shifting the K^+^-equilibrium potential to a more positive level, consequently inducing depolarization of the cell. As schematically illustrated in Figure 2, KCl-induced depolarization should cause a conformational change in the sensor, thus bringing donor and acceptor fluorochromes into proximity. The resulting emission-free energy transfer (FRET) than results in an increase in acceptor and a simultaneous decrease in donor fluorescence intensity (Figure 2a,b).

No change in Mermaid emission ratio was observed under control conditions, i.e., addition of Hepes Ringer (Figure 2c). In contrast, Mermaid acceptor/donor emission ratio raised significantly upon KCl stimulation (50 mM) when compared to baseline (before KCl application, Figure 2d), thereby depicting general functionality of Mermaid in primary mouse PASMCs.

### 3.3. Potassium–Chloride Dose-Dependently Enhances Mermaid’s FRET Ratio

**Figure 3:**
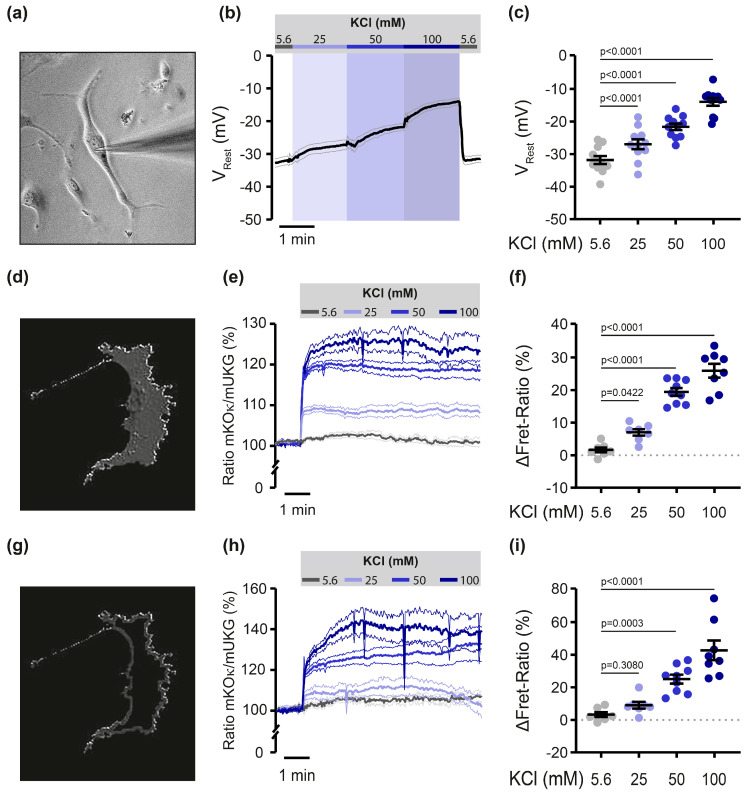
Mermaid FRET ratio dose-dependently increases in response to KCl. (**a**) Representative picture of a primary mouse PASMC that is attached to a patch clamp pipette during whole-cell recording. (**b**) Original voltage traces of patch clamp experiments in PASMCs, exposed to increasing doses of KCl. KCl concentrations in mM as indicated. A 5.6 mM KCl is already contained in the Hepes Ringer and therefore serves as control condition. Data are shown as mean (solid line) ± SEM (narrow lines). *n* = 11 experiments, i.e., individual cells. (**c**) Statistical analysis of data shown in panel (**b**). (**d**) Representative ratio image showing a whole PASMC. (**e**) Acceptor/donor FRET ratio (in %) of PASMCs challenged with different KCl concentrations as indicated. Traces were overlayed for better comparison. Data are shown as mean (solid line) ± SEM (narrow lines). *n* = 7–9 experiments per condition. (**f**) Statistical analysis of data shown in panel (**e**): Delta (∆) of FRET ratio of whole PASMC after KCl-application, depicted as mean ± SEM. (**g**) Representative image showing the membrane rim of the same PASMC as shown in panel (**d**) and the (**h**) corresponding traces and (**i**) statistical analysis.

**Figure A1:**
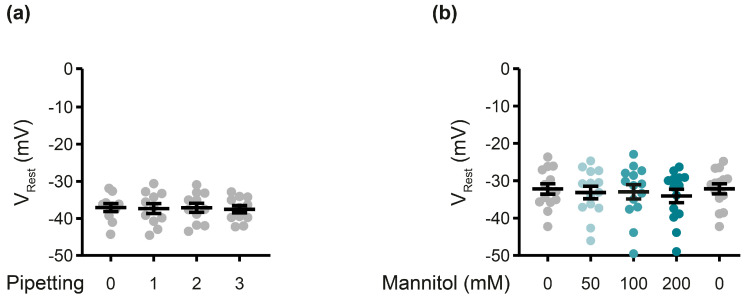
Membrane potential of mouse PASMCs is not affected by pipetting, time or osmolality. (**a**) PASMCs were subjected to pipetting maneuvers using the same time intervals, velocity and volume as those in the KCl experiments depicted in Figure 3b,c. Instead of KCl, Hepes Ringer was applied three times. (**b**) Increasing osmolality by elevating doses of mannitol, equivalent to the increase in osmolality induced by KCl in Figure 3b,c, had no effect on membrane potential of mouse PASMCs.

Following validation of Mermaid’s general functionality in primary PASMCs, the sensor was finally tested for its capability to detect small dose-dependent changes in membrane potential. Therefore, PASMCs were challenged with increasing extracellular KCl concentrations, while the membrane potential was measured in classical whole-cell recordings via the patch clamp technique (Figure 3a–c). As shown in Figure 3a–c, KCl increased membrane potential by 4.8 ± 0.6 mV (25 mM KCl), 10.1 ± 0.7 mV (50 mM KCl) and 17.7 ± 1.5 mV (100 mM KCl), thus representing the range in membrane voltage changes observed in PASMCs under physiological conditions. As controls, PASMCs underwent pipetting maneuvers mirroring the timing, velocity and volume of the KCl experiments, confirming no experimental impact on PASMC membrane potential (see Appendix A, Figure A1a). Additionally, elevating osmolality with mannitol doses equivalent to those induced by KCl (Figure 3b,c) also showed no effect on cellular membrane potential (see Appendix A, Figure A1b).

**Figure A2:**
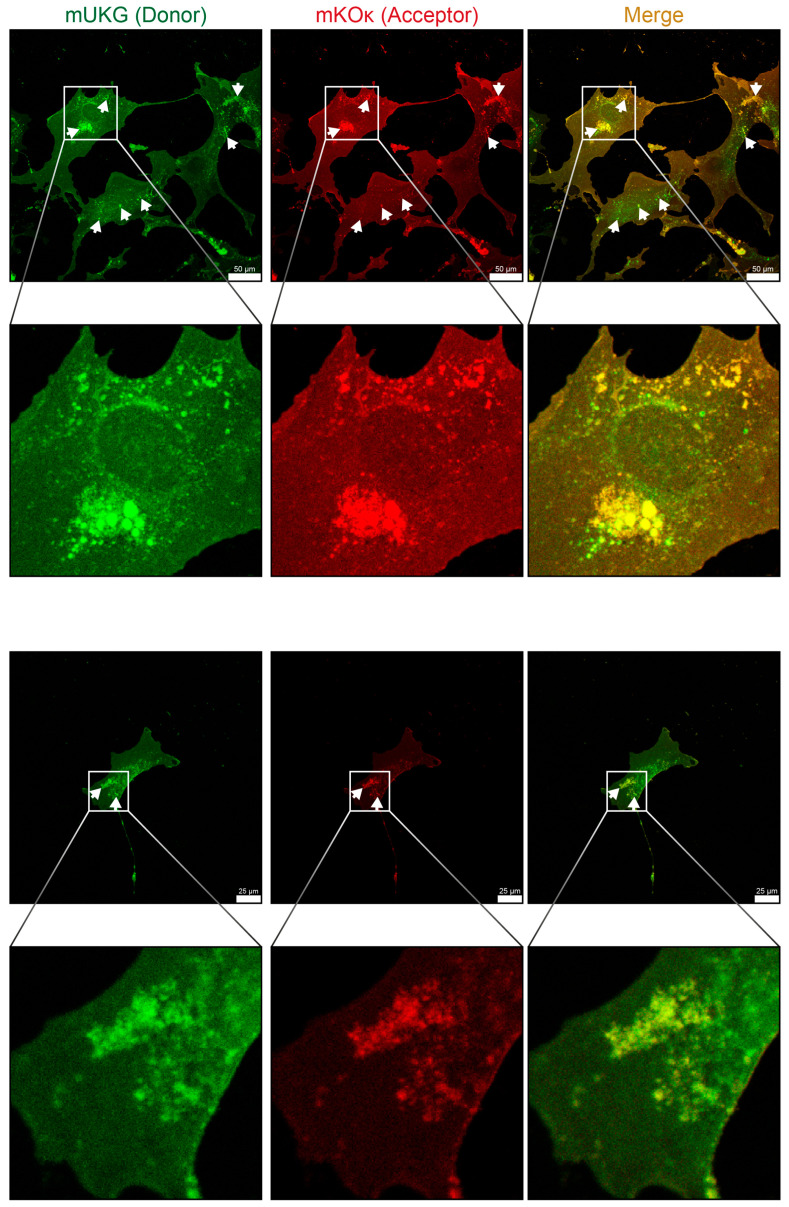
Expression pattern of Mermaid in pulmonary arterial smooth muscle cells. Confocal fluorescence images of PASMCs transduced with Mermaid revealed Mermaid-associated non-specific signals (exemplarily highlighted with white arrows) in both the donor (mUKG, green) and the acceptor channel (mKO_K_, red) as well as displayed by the yellow color in the merged image. Scale bar: 50 µm (**top panel**) and 25 µm (**lower panel**).

Similar to patch clamp experiments, KCl induced a dose-dependent increase in FRET ratio for all three concentrations when analyzing the fluorescence signal of the complete cells (Figure 3d–f and Table 1). However, Mermaid has already been shown to label some unspecific cellular structures [31,38,39]. In this regard, Tian and co-workers demonstrated that around 17% of the fluorescence signal of Mermaid-expressing ventricular myocytes originates from structures that are not connected to the extracellular space and thus do not contribute to the voltage-dependent changes of the Mermaid signal [31]. In line with these reports, we also observed Mermaid-expressing PASMCs to partly show fluorescent aggregates (see Appendix A, Figure A2, white arrows), potentially originating from the described aggregate formation, non-specific Mermaid expression in organelle membranes or intrinsic cellular fluorescence. To better distinguish membrane-derived from unspecific intracellular FRET signals, we chose an additional approach in which only the outer plasma membrane edge of PASMCs was analyzed, while the main soma of the cell was omitted (Figure 3g,h). Comparable to whole-cell analysis, a depolarizing stimulus with 50 mM as well as 100 mM KCl elicited a significant FRET ratio increase compared to baseline, while no change in FRET emission rate was visible for the mildest depolarizing stimulus, i.e., 25 mM KCl (Figure 3i and Table 1).

## 4. Discussion

The assessment of changes in membrane potential, particularly its nuanced alterations, is of crucial importance in various (patho)physiological contexts. For instance, it has been widely recognized for decades that only subtle changes of 5 to 15 mV in the membrane potential of pulmonary arterial smooth muscle cells (PASMCs) plays a crucial role in initiating hypoxic pulmonary vasoconstriction, which is necessary for maintaining adequate gas exchange as well as the development and progression of pulmonary hypertension, a life-threatening and currently incurable disease. Consequently, substantial efforts have been made to understand the intricate relationship between PASMC membrane potential and cellular and vascular reactivity, respectively. Despite these considerable efforts over many years, to the best of our knowledge, no study has reported the use of a fluorescence-based voltage sensor in PASMCs. Since a fluorescence-based voltage sensor would represent a more user-friendly and more widely applicable alternative to the classical patch clamp technique, the aim of the current study was to test whether a fluorescence-based voltage sensor (a) can be functionally expressed in PASMCs and (b) can serve as a reliable alternative to detect subtle changes in membrane potential of these cells. However, most (if not all) currently available fluorescence-based voltage sensors are designed to detect changes in membrane potential of excitable cells, such as neuronal cells or cardiomyocytes that are characterized by amplitudes of up to 120 mV. By their nature, action potentials are defined not only by this extremely high amplitude but also by their rapid velocity, occurring within milliseconds. Consequently, recent advancements in the development of fluorescence-based voltage sensors have primarily focused on enhancing their temporal and spatial resolution as well as their signal size [40] rather than focusing on detecting subtle changes in membrane potentials of a few millivolts (which are uncommon in excitable cell types). As a result, the characterization of these (and any newly developed) sensors is commonly conducted using much higher voltage amplitudes, i.e., using hyper- and depolarizing steps of a minimum of 20 mV or above [41,42,43,44,45,46,47]. As a result, to the best of our knowledge, in addition to the lack of reports about voltage sensors in PASMCs, there is currently no fluorescence-based voltage sensor described that reliably detects such small changes in membrane potential, not even in different cell types. Therefore, we tested whether the genetically encoded FRET sensor Mermaid is applicable in precapillary PASMCs to detect small changes in membrane potential in these cells.

Mermaid was developed in 2008 with the aim of generating a voltage sensor that enables precise spatial and temporal visualization of action potentials in cultured excitable cells [24]. Consequently, Mermaid’s ability to detect strong changes in membrane potential has been successfully evaluated in cardiac [30,31] and neuronal [24,28,29] cells. Besides its use in vitro, Mermaid was also used for depicting voltage changes in the beating zebrafish heart in vivo [30]. However, until now, there has been no documented utilization of Mermaid in pulmonary cell types, PASMCs in particular. In addition, while Mermaid has been established as a reliable tool for depicting strong changes in membrane potentials, its capability to recognize subtle changes in membrane potential, such as those physiologically occurring in PASMCs [2,3,18,33], remained unexplored. Given that Mermaid could serve as a feasible alternative whenever classical patch clamp is not available, our objective was to test whether Mermaid (i) can be effectively expressed in primary mouse PASMCs and (ii) provides reliable measurements of subtle voltage changes in these cells.

Upon lentiviral transduction, FRET sensor Mermaid showed robust expression and functionality in the plasma membrane of precapillary mouse PASMCs. To elucidate the capability of Mermaid to detect subtle changes in PASMCs, we used extracellular KCl as a depolarizing stimulus and measured the resulting change in membrane potential by classical whole-cell patch clamp recordings. In response to KCl, we observed changes in membrane potential by 4.8 ± 0.6 mV (25 mM KCl), 10.1 ± 0.7 mV (50 mM) and 17.7 ± 1.5 mV (100 mM), all of which are in the range of membrane potential changes that physiologically occur in PASMCs [2,3,18,33].

Upon a strong depolarizing stimulus with 100 mM KCl (**≙** 17.7 ± 1.5 mV), Mermaid displayed a FRET ratio change of 25.9 ± 2.1%. This result outperforms even with the formerly reported ~40% FRET ratio change upon a much stronger depolarization (100 mV) in *Xenopus laevis* oocytes [24]. Of note, even with less depolarizing stimuli using 25 mM and 50 mM KCl, significant changes in FRET ratio were observed in our study. The sensor response to KCl was found to be linear at least in the three KCl concentrations tested, which can be regarded as a major advantage of this voltage sensor.

However, despite its primary localization in the plasma membrane, Mermaid formed certain unspecific intracellular aggregates. This observation is in line with previous reports: Tian and co-workers demonstrated that approximately 17% of the fluorescence signal does not originate from structures connected to the extracellular space and therefore does not contribute to voltage-dependent changes in the Mermaid signal [31]. For that reason, we developed a secondary analysis approach focusing on the outermost plasma membrane rim only. This analysis also proves significant FRET ratio changes for 50 mM as well as 100 mM KCl. Since physiologically only small voltage changes of 5–15 mV occur in PASMCs [2,3,18,33], the lowest KCl stimulus (25 mM) was of greatest importance for our study. Unfortunately, when analyzing the membrane rim only, Mermaid was not able to depict a significant change in fluorescence emission. Thus, it precludes this FRET imaging approach to further decipher subtle physiological changes in membrane potential in PASMCs. However, both analyzing approaches (whole-cell and membrane-only) are feasible in principle, provided one is aware of the associated uncertainties regarding the observed non-specific signals in whole-cell analysis and exercises extreme caution in interpreting the data obtained with this approach.

Although Mermaid could well be a suitable alternative for applications that resolve changes in membrane potential of more than 10 mV, one should be aware of some further disadvantages, which also partially apply to many—if not all—genetically encoded voltage indicators (GEVIs).

As previously elucidated by Tian et al. [31], the presence of motion artefacts—a hallmark of contractile cells such as PASMCs—can negatively influence the Mermaid-based FRET approach. In the chosen ‘membrane-only’-analysis, we observed that motion artefacts sometimes even precluded a valid analysis. As stated by Tian et al., pharmacological intervention might be suitable to suppress these motion artefacts but runs the risk of introducing undesired downstream effects through the manipulation of cellular signal transduction pathways.

A further disadvantage of Mermaid is the prerequisite for lentiviral transduction. Lentiviral vectors are considered safe, with minimal immunogenicity, while enabling stable integration of genes into the host genome [48,49]. The resulting permanent and controlled gene expression is of advantage compared to other viral vectors, such as AAVs that may induce high initial gene expression that decreases over time or become cytotoxic due to overwhelming the cell with excessive protein production [48,50]. However, the introduction of a viral vector and an external protein carries the inherent risk of modifying cellular physiology, thereby potentially disrupting subsequent cellular downstream pathways or pharmacological interventions intended to elucidate molecular pathways in cellular response. Besides the necessity of genetic manipulation and the associated stress of viral infection, only cultured cells may be investigated with Mermaid. Longer-lasting cell culture and passaging of freshly isolated SMCs are often associated with a phenotype switch from a highly specialized contractile phenotype to a less differentiated state with increased capacities for motility, protein synthesis and proliferation [51]. Since lentiviral transduction and subsequent overexpression of an extrinsic protein requires cell culture periods lasting at least several days, such a phenotype switch and an associated change in the properties compared to freshly isolated PASMCs cannot be completely excluded in Mermaid experiments.

Moreover, Mermaid only allows for detecting relative changes in membrane potential. In contrast to classical patch clamp recordings, a Mermaid-based approach lacks the capability to determine the exact value of the membrane potential or to quantify the amplitude of hyper- or depolarization. This consequently implies that whenever it is crucial to ascertain the specific value of the membrane potential, such as determining whether the threshold for gating of a particular voltage-dependent ion channel is reached, a Mermaid-based approach is not suitable. Nevertheless, if the sole concern is detecting the presence or absence of a change in membrane potential, Mermaid could serve as a viable alternative to patch clamp recordings.

Another limitation of Mermaid might be its detection range, which could possibly lead to limitations when investigating hyperpolarizing agents, such as NO or K-ATP-openers. Considering that the structure of Mermaid is based on two fluorochromes approaching each other during depolarization, it conversely implies that they move apart during hyperpolarization, thus leading to a weakening signal and potentially to complete signal loss. This suggests that there is a detection limit to negative membrane potential that cannot be surpassed. Based on these findings, the detection of hyperpolarization crucially relies on the cellular resting membrane potential and thus may only be detectable to a certain extent, or within a specific range. In accordance, Tsutsui et al. reported a lower detection limit for the FRET signal of approximately −60 mV in *Xenopus* oocytes and NeuroA2 cells [24]. Given that PASMCs exhibit a more positive membrane potential and are even inherently depolarized in pulmonary hypertension [52], it is plausible that the membrane potential of PASMCs would still be within the described detection limit of Mermaid, even in response to a hyperpolarizing agent. For this reason, we consider this sensor suitable for use in PASMCs for both depolarizing and hyperpolarizing agents, but it may limit its application in other cell types characterized by more negative resting membrane potentials.

## 5. Conclusions

In our study, we found Mermaid to be a suitable alternative for reliably detecting qualitative membrane voltage changes of more than 10 mV in primary mouse PASMCs, whenever classical patch clamp is not available. However, one should be aware of the limitations associated with this FRET-based approach.

It is noteworthy that the drawbacks highlighted for Mermaid extend beyond this particular voltage sensor and are at least partially relevant to all GEVIs. Nevertheless, exploring alternative GEVIs in future studies may prove valuable, potentially emphasizing the already high sensitivity of Mermaid or identifying options with even greater responsiveness. In this context, however, it is important to acknowledge the difficulties with transfecting primary cells such as PASMCs, suggesting that not all GEVIs may be appropriate for this cell type.

## Figures and Tables

**Table 1:** Mean values of patch clamp and FRET experiments as shown in Figure 3.

	Patch Clamp			Whole Cell			Membrane Only		
KCl (mM)	V_Rest_ (mV)	SEM	*n*	∆FRET- Ratio (%)	SEM	*n*	∆FRET- Ratio (%)	SEM	*n*
5.6	−31.94	1.228	11	1.629	0.742	7	3.073	1.434	7
25	−27.13	1.493	11	7.003	1.004	7	8.745	2.076	7
50	−21.82	0.991	11	19.43	1.223	9	24.73	2.735	9
100	−14.20	1.186	11	25.95	2.116	8	42.23	5.991	8

KCl: Potassium Chloride, V_Rest_: Resting membrane potential; SEM: standard error of the mean; *n*: number of experiments on individual cells.

## Data Availability

The data presented in this study are available on reasonable request from the corresponding author.
